# Impact of the COVID-19 Pandemic on Survival in the Patients With the Intra-Abdominal Infections

**DOI:** 10.3389/fmed.2021.687415

**Published:** 2021-10-20

**Authors:** Lydia Gálvez-Benítez, Ángel Rodríguez-Villodres, Rocío Álvarez-Marín, Rosa Jiménez-Rodríguez, José Antonio Lepe-Jiménez, Jerónimo Pachón, Younes Smani

**Affiliations:** ^1^Clinical Unit of Infectious Diseases, Microbiology and Preventive Medicine, Virgen del Rocío University Hospital, Seville, Spain; ^2^Institute of Biomedicine of Seville (IBiS), Virgen del Rocío University Hospital/CSIC/University of Seville, Seville, Spain; ^3^Department of General and Digestive Surgery, Virgen del Rocío University Hospital, Seville, Spain; ^4^Department of Medicine, University of Seville, Seville, Spain

**Keywords:** COVID-19, intra-abdominal infection, *Escherichia coli*, surgery, mortality

## Abstract

**Objective:** To analyze the availability and access to the hospital for the patients with intra-abdominal infections (IAIs) by *Escherichia coli (E. coli)* as a result of the coronavirus disease 2019 (COVID-19) pandemic and the impact of these changes in the diagnosis and their effects on the death of these patients.

**Methods:** Two prospective observational cohorts of the patients with IAI by *E. coli* were conducted in 2016 (the pre-COVID-19, *n* = 108) and in 2020 (during the COVID-19, *n* = 96) at the University Hospital of Seville, Spain. The demographic and clinical variables of the patients were collected and analyzed. The patients were followed-up for 120 days, until the hospital discharge or death. The bivariate and multivariate analyses were performed.

**Results:** Both the cohorts were homogeneous according to age, sex, emergency surgery cause, immunosuppression, neutropenia, acquisition type, and previous intervention. The patients attended during the COVID-19 had significantly higher Charlson comorbidity index and the more McCabe score, required more emergency surgery, had more severe infections with the higher rates of septic shock and sepsis, and the presence of additional care support such as a nasogastric tube. They were diagnosed later; the time intervals between the symptoms onset (SO) to the first medical contact or surgical intervention (SI) and between the first medical contact to the admission or SI were significantly higher. The death rates during the COVID-19 and the pre-COVID-19 were 16.7 and 6.5%, respectively (*p* = 0.02). Finally, the multivariate analysis in both the cohorts together identified the patients diagnosed during the COVID-19, the longer period from SO to SI, septic shock, and the Charlson comorbidity index as the independent factors associated with death.

**Conclusion:** This study showed the impact of the COVID-19 pandemic on the clinical outcome and death due to IAI with an extension of the time between SO and SI.

## Introduction

The coronavirus disease 2019 (COVID-19) pandemic continues to be an important threat to health due to its direct effects and the difficulties to attend to the other diseases (1). Significant reduction in the admission of patients with other diseases (cancer, cardiovascular disease, and stroke) and delay in their diagnosis has been detected in Spain and in other European, American, and Asian countries (2–5). Consequently, higher numbers of death in patients with these diseases have been reported during the COVID-19 pandemic (2, 4).

It is well-known that the management of patients with intra-abdominal infections (IAIs) is increasingly complicated due to the lack of the optimal therapeutic options, which boost the risk of therapeutic failure and mortality, especially in the patients with the secondary bloodstream infection, when they developed sepsis or septic shock (6).

The aim of this study was to analyze the availability and access to the hospital for the patients with IAI as a result of the COVID-19 pandemic, the impact of these changes in the diagnosis, and their effects on the death of the patients diagnosed with IAI due to *Escherichia coli (E. coli)*, as its most frequent etiology.

## Materials and Methods

### Study Design, Patients, and Ethics

This is a *post-hoc* analysis of the two prospective observational cohorts from February 1 to November 30, 2016, and from February 1 to November 30, 2020, respectively, recruited at the Virgen del Rocío University Hospital (Seville, Spain). About 108 and 96 adult patients (≥18 years) with IAI by *E. coli* isolated from the intra-abdominal samples or blood cultures were selected for both the cohorts, respectively. Only the first IAI episode was included in this study during the admissions of the patient. The pathogenic role of *E. coli* isolated from the intra-abdominal samples, as colonization or infection, was evaluated by a member of the Infectious Disease Services and/or the intensive care unit, according to the previously defined criteria (7). The study was approved by the Ethics Committee of the Hospital Universitario Virgen Macarena and the Virgen del Rocío University Hospital (approval no. 0023-N-16 and 0088-N-20). The written informed consent was signed by all the patients before inclusion in the cohorts.

### Variables

The following variables were collected from the patients: age and sex; patients admitted during the COVID-19 pandemic period; emergency surgery; bacteremia; comorbidities and severity of the chronic underlying diseases (the Charlson comorbidity index and the McCabe score) (8, 9); presence of solid or the hematological malignancies, immunosuppression, and neutropenia (<500 neutrophils/μl); acquisition type (defined as a community acquired, healthcare acquired, or hospital acquired), polymicrobial infection, sepsis, or septic shock (10); the presence of the devices (urinary catheter, transitory or permanent central venous catheter, nasogastric tube, and mechanical ventilation); and the time intervals between the symptoms onset (SO) and the surgical intervention (SI).

The Charlson comorbidity index is a score that predicts the 1-year mortality. It was calculated according to the 19 comorbid conditions of patients, including diabetes with diabetic complications, congestive heart failure, peripheral vascular disease, chronic pulmonary disease, mild and severe liver disease, hemiplegia, renal disease, leukemia, lymphoma, metastatic tumor, and acquired immunodeficiency syndrome (8, 11). The severity of comorbidity was categorized into the three grades: mild, with Charlson comorbidity index score of 1–2; moderate, with Charlson comorbidity index score of 3–4; and severe, with Charlson comorbidity index score of ≥ 5 (11).

The McCabe score is used to predict the likelihood for the survival of the patients with Gram-negative bacteremia (9). It classifies all the hospitalized patients into the three categories: nonfatal (score = 0, patient survival > 5 years), ultimately fatal (score = 1, patient survival 1–4 years), and rapidly fatal (score = 2, patient survival < 1 year).

The patients were followed-up for 120 days, until the hospital discharge or death, whichever occurred first, and the clinical decisions were made by the physicians in charge of the patients. All the data of the patients were collected from the electronic medical record and the time intervals were collected from the anamnesis of the admission fact sheet of the patient.

### Microbiological Studies

The *E. coli* isolates were identified by using the matrix-assisted laser desorption ionization time-of-flight (MALDI-TOF) mass spectrometry (Bruker Daltonik GmbH, Leipzig, Germany). Extended-spectrum ß-lactamases (ESBLs) production was determined by using the broth microdilution synergy test according to the European Committee on Antimicrobial Susceptibility Testing (EUCAST) standards (12).

### Statistical Analysis

The primary endpoint was the occurrence of death at the end of follow-up as the dependent variable. A descriptive analysis was performed for the demographics and clinical variables, with the median and interquartile range for the quantitative variables and the frequency distribution for the qualitative variables. The Fisher's exact test and the chi-squared test were used for the categorical variables and the continuous variables were analyzed by using the Mann–Whitney *U*-test or the Student's *t*-test. To examine the factors associated with death, the univariable logistic regression analysis was performed in the combined dataset of both cohorts (*n* = 204 patients). To identify the factors independently associated with death, the multivariable logistic regression model was built, including those variables associated with the dependent variable in the bivariate analysis (*p* < 0.05), after analyzing them to avoid the collinearity and their presence at the inclusion of the patients in the cohorts. With respect to the period times, this study included the days from the SO to intervention because it is the most accurate period. The goodness of fit for the logistic regression was assessed by following the Hosmer–Lemeshow test, with a chi-square of 8.3 in the last step of the regression and with *p* = 0.403. The survival curves were obtained by using the Kaplan–Meier method and were compared by using the log-rank tests. The differences were considered as significant at *p* < 0.05. All the statistical analyses were performed by using the SPPS software package (version 25.0, IBM Corporation, Armonk, New York, USA).

## Results

### Demographics, Clinical, and Microbiological Characteristics of the Patients With IAI in the Pre-coronavirus Disease 2019 Pandemic Period and During the COVID-19 Pandemic Period

A total of 204 patients with IAI by *E. coli* were included in both cohorts. The demographics and clinical features of both the cohorts were grouped together and their differences between both the cohorts are summarized in Table 1. Both the IAI cohorts were homogeneous according to the demographics (age and sex), cause of emergency surgery, immunological state (immunosuppression or neutropenia), acquisition type, and previous intervention. The patients with IAI attended during the COVID-19 pandemic had more comorbidities, with the higher Charlson comorbidity index (*p* < 0.001) and more frequent ultimately or the rapidly fatal McCabe score (*p* < 0.001). The patients during the COVID-19 pandemic required more emergency surgery (*p* < 0.001) and had more severe infections, with higher rates of septic shock (*p* = 0.001), sepsis (*p* < 0.001), and the presence of additional care support such as nasogastric tube (*p* = 0.02). In contrast, a lower percentage of the patients with bacteremia was also observed during the COVID-19 pandemic. The blood cultures were requested by the physicians only in 26% of the patients in the COVID-19 pandemic period compared with 72% of the patients in the pre-COVID-19 pandemic period. However, the rates of the positive blood cultures were similar in both the pandemic periods (15.4 and 12%, respectively).

**Table 1:** Demographics, clinical, and microbiological variables of the patients with IAI in the pre-COVID-19 pandemic period and during the COVID-19 pandemic period.

**Factor**	**IAI patients** **(*n* = 204)**	**IAI patients in pre-COVID-19 period** **(*n* = 108)**	**IAI patients during COVID-19 period** **(*n* = 96)**	***P*-value**
Age, median (range)	64 (52.8–73)	64.5 (50.7–73.2)	64.0 (54.7–72.2)	0.83
Male sex, No. (%)	120 (58.8)	70 (64.8)	50 (52.1)	0.06
IAI patients in COVID-19 period, No. (%)	96 (47.0)	NA	96 (100)	NA
Emergency surgery, No. (%)	94 (46.1)	36 (33.3)	58 (60.4)	**<0.001**
Post-surgical abscess	60 (29.4)	5 (4.6)	9 (9.4)	0.82
Post-surgical peritonitis	22 (10.8)	6 (5.6)	7 (7.3)	0.53
Cholecystitis	49 (24.0)	8 (7.4)	16 (16.7)	0.56
Appendicitis	21 (10.3)	10 (9.2)	9 (9.4)	0.15
Hollow viscus perforation	23 (11.3)	6 (5.6)	11 (11.4)	0.77
Diverticulitis	10 (4.9)	1 (0.9)	4 (4.1)	0.38
Others	19 (9.3)	0 (0.0)	2 (2.1)	NA
Bacteremia, No. (%)	15 (7.3)	12 (11.1)	3 (3.1)	**<0.02**
McCabe score, ultimately or rapidly fatal	56 (27.4)	19 (17.8)	37 (38.5)	**<0.001**
Charlson index, median (range)	2 (0–5)	2 (0–3)	4 (2–6)	**<0.001**
**Comorbid condition, No. (%)**				
Cancer	64 (31.4)	28 (25.9)	36 (37.5)	0.07
Metastatic cancer	16 (7.8)	11 (10.2)	5 (5.2)	0.18
Diabetes	30 (14.7)	13 (12.0)	17 (17.7)	0.25
Autoimmune diseases	5 (2.4)	1 (0.9)	4 (4.2)	0.14
Dementia	1 (0.5)	1 (0.9)	0 (0.0)	0.34
Chronic lung disease	19 (9.3)	9 (8.3)	10 (19.4)	0.61
Vascular disease	8 (3.9)	5 (4.6)	3 (3.1)	0.58
Chronic kidney disease	7 (3.4)	5 (4.6)	2 (2.1)	0.31
Liver disease	8 (3.9)	5 (4.6)	3 (3.1)	0.58
Hematologic malignancies	2 (1.0)	1 (0.9)	1 (1.0)	0.36
Immunosuppression	20 (9.8)	12 (11.1)	8 (8.3)	0.50
Neutropenia	1 (0.5)	0 (0.0)	1 (1.0)	0.29
**Acquisition type, No. (%)**				
Community-acquired	115 (56.4)	59 (54.6)	56 (58.3)	0.59
Healthcare-related	16 (7.8)	8 (7.4)	8 (8.3)	0.80
Hospital-acquired	73 (35.8)	41 (38.0)	32 (33.3)	0.49
Polymicrobial infection, No. (%)	110 (53.9)	61 (56.5)	49 (51.0)	0.44
Septic shock, No. (%)	21 (10.3)	4 (3.7)	17 (17.7)	**0.001**
Sepsis, No. (%)	43 (21.1)	13 (12.0)	30 (31.2)	**<0.001**
Previous intervention, No. (%)	72 (35.3)	41 (38.3)	31 (32.3)	0.37
**Presence of devices, No. (%)**				
Urinary catheter	121 (59.3)	58 (53.7)	63 (65.6)	0.08
Transitory CVC	73 (35.8)	40 (37.0)	33 (34.4)	0.69
Permanent CVC	2 (1.0)	0 (0.0)	2 (2.1)	0.13
Nasogastric tube	63 (30.9)	26 (24.1)	37 (38.5)	**0.02**
Mechanical ventilation	34 (16.7)	13 (12.0)	21 (21.9)	0.05
**Time intervals between the SO and the intervention, median of days (range)**				
From SO to 1^st^ medical contact	1 (0–3.5)	0 (0–2)	2 (0–7)	**0.002**
From SO to intervention	6 (2–16)	4 (1–8)	7.5 (4–32)	**<0.001**
From 1^st^ medical contact to admission	1 (0–3.5)	2 (0–5)	6 (1–16)	**<0.001**
From 1^st^ medical contact to intervention	3 (1–10)	0 (0–1)	4.5 (1–11.5)	**<0.001**
From admission to intervention	1 (0–5)	1 (0–4)	1 (1–7)	0.186
Appropriate empirical therapy, No. (%)	187 (91.7)	96 (88.9)	91 (94.8)	0.13
Death, No. (%)	23 (11.3)	7 (6.5)	16 (16.7)	**0.02**

*COVID-19, coronavirus disease 2019; IAI, intra-abdominal infection; CVC, central venous catheter; SO, symptoms onset; NA, not applicable*.

*Values in bold indicate that P-value is inferior to 0.05*.

**Figure 1:**
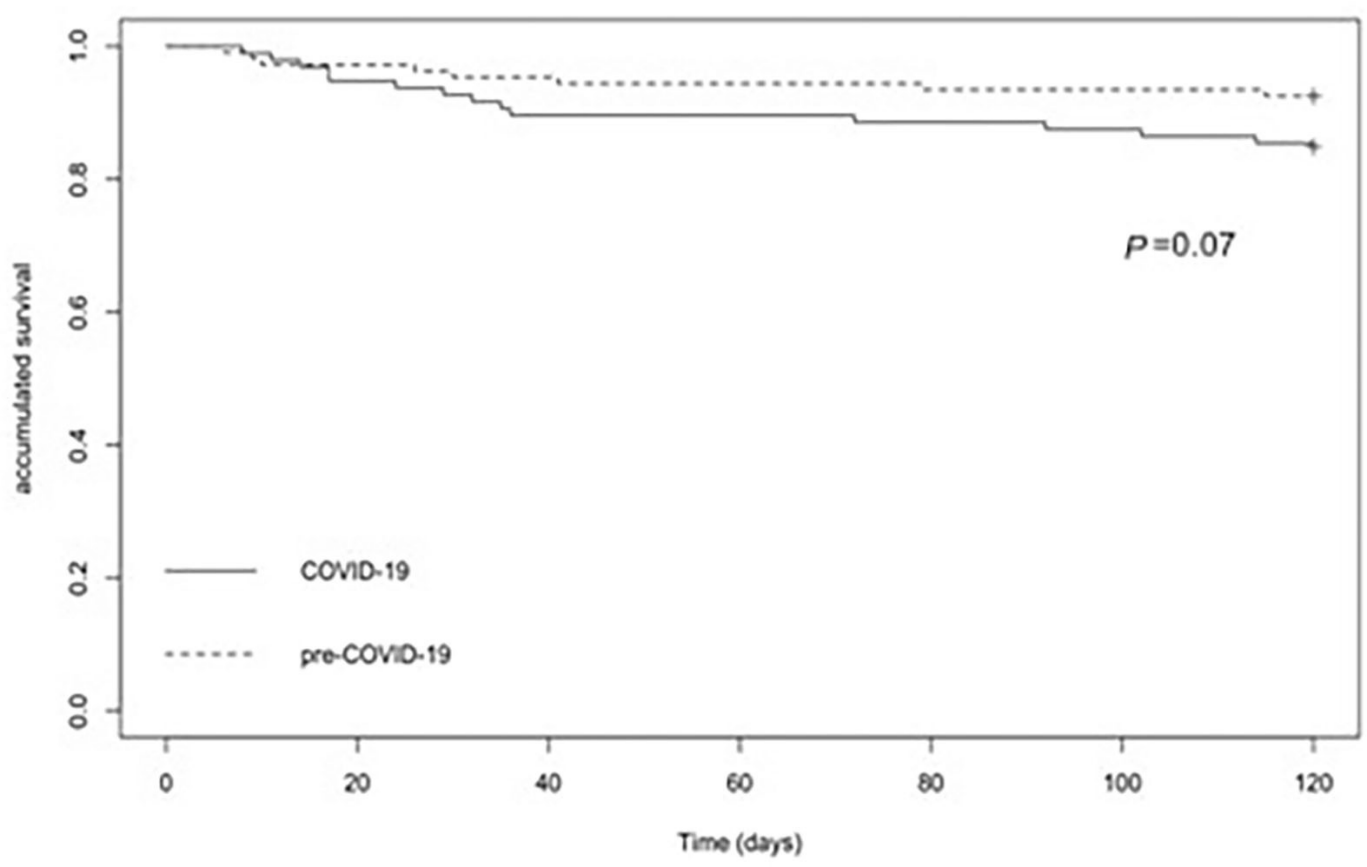
The Kaplan–Meier plots among the patients with the intra-abdominal infections (IAI) during the pre-coronavirus disease 2019 (COVID-19) pandemic period and the COVID-19 pandemic period.

The patients with IAI during the COVID-19 were diagnosed later; time intervals between the SO to the first medical contact were higher (*p* = 0.002) or to the SI were near two times (*p*< 0.001) more than for the patients with IAI of the pre-COVID-19 cohort (Table 1). Similarly, the day intervals between the first medical contact and the admission or the SI were equal or higher than the three times, respectively, for the patients with IAI in the 2020 cohort vs. for the patients with IAI in the 2016 cohort (*p*< 0.001 and *p*< 0.001, respectively, for the admission and SI). With respect to the death rates, 16.7 and 6.5% of the patients died in the 2020 and 2016 cohorts, respectively (*p* = 0.02). The Kaplan–Meier curves for the survival of the patients with IAI in the pre-COVID-19 pandemic cohort and during the COVID-19 pandemic cohort are shown in Figure 1. Overall, although both the curves showed different declines, these differences were not statistically significant (*p* = 0.07). Moreover, the rate of ESBL production was higher during the COVID-19 pandemic cohort compared to the pre-COVID-19 pandemic cohort (9.4 vs. 2.8%, respectively; *p* = 0.04). Although, the rate of the appropriate empirical therapy was similar in both cohorts (94.8 vs. 88.9%, respectively; *p* = 0.13) (Table 1).

### Bivariate and Multivariate Analyses of the Factors Related to Death in the Pre-COVID-19 Pandemic Cohort and the COVID-19 Pandemic Cohort Grouped Together

The analysis of the factors related to death in both the cohorts grouped together is summarized in Table 2. The bivariate analysis showed that the dead patients are older (*p* = 0.02); more frequently admitted during the COVID-19 pandemic period (*p* = 0.02); had more comorbidities (Charlson comorbidity index, *p*< 0.001); more frequent ultimately or the rapidly fatal McCabe score (*p* < 0.001); required more emergency surgery (postsurgical peritonitis and hollow viscus perforation, *p* = 0.02 and *p* = 0.01, respectively); and had more severe infections, with the higher rates of bacteremia (*p* < 0.001), septic shock (*p* < 0.001), sepsis (*p* < 0.001), and the presence of additional care support such as urinary catheter (*p* < 0.001), transitory central venous catheter (CVC) (*p* < 0.001), nasogastric tube (*p* < 0.001), and mechanical ventilation (*p* < 0.001). In contrast, a higher percentage of the patients with the polymicrobial infection and appropriate empirical therapy in the first 24 h was observed in the survival group (*p* = 0.01 and *p* < 0.001, respectively). With respect to the time intervals between the SO and the SI, the dead patients have diagnosed later; the time intervals between the SO to the SI and between the first medical contact and the SI were near and higher to the two times (*p* = 0.03 and *p* = 0.04) for the dead patients vs. survival patients (*p* = 0.03 and *p* = 0.04), respectively.

**Table 2:** Univariate analysis of the factors related to death in a combined cohort of the pre-COVID-19 pandemic cohort and the COVID-19 pandemic cohort.

**Factor**	**Death** **(*n* = 23)**	**Survival** **(*n* = 181)**	***P*-value**
Age, median (range)	72 (62–76)	63 (51–73)	**0.02**
Male sex, No. (%)	14 (60.9)	106 (58.6)	0.83
IAI patients in COVID-19 period, No. (%)	16 (69.6)	80 (44.2)	**0.02**
Emergency surgery, No. (%)	13 (56.5)	81 (44.7)	0.28
Post-surgical abscess	5 (21.7)	55 (30.4)	0.39
Post-surgical peritonitis	6 (26.1)	16 (8.8)	**0.02**
Cholecystitis	2 (8.7)	47 (26.0)	0.07
Appendicitis	1 (4.3)	20 (11.0)	0.31
Hollow viscus perforation	6 (26.1)	17 (9.4)	**0.01**
Diverticulitis	0 (0)	10 (5.5)	0.24
Others	3 (13.0)	16 (8.9)	0.51
Bacteremia, No. (%)	6 (26.1)	9 (5.0)	**<0.001**
McCabe score, ultimately or rapidly fatal	16 (69.6)	40 (22.1)	**<0.001**
Charlson index, median (range)	6 (4–7.5)	2 (0–4)	**<0.001**
**Comorbid condition, No. (%)**			
Cancer	9 (39.1)	55 (30.4)	0.39
Metastatic cancer	3 (13.0)	13 (7.2)	0.39
Diabetes	3 (13.0)	27 (14.9)	1.00
Autoimmune diseases	1 (4.3)	4 (2.2)	0.45
Dementia	0 (0)	1 (0.6)	1.00
Chronic lung disease	3 (13.0)	16 (8.8)	0.51
Vascular disease	1 (4.3)	7 (3.9)	1.00
Chronic kidney disease	3 (13.0)	4 (2.2)	**0.03**
Liver disease	3 (13.0)	5 (2.8)	**0.01**
Hematologic malignancies	0 (0)	2 (1.1)	0.88
Immunosuppression	4 (17.4)	16 (8.9)	0.25
Neutropenia	1 (4.3)	0 (0)	0.11
**Acquisition type, No. (%)**			
Community-acquired	10 (43.5)	105 (58.0)	0.19
Healthcare-related	3 (13.0)	13 (7.2)	0.40
Hospital-acquired	10 (43.5)	63 (34.8)	0.41
Polymicrobial infection, No. (%)	7 (30.4)	103 (56.9)	**0.01**
Septic shock, No. (%)	7 (30.4)	14 (7.7)	**<0.001**
Sepsis, No. (%)	16 (69.6)	27 (14.9)	**<0.001**
Previous intervention, No. (%)	7 (30.4)	14 (7.7)	0.39
**Presence of devices, No. (%)**			
Urinary catheter	21 (91.3)	100 (55.2)	**<0.001**
Transitory CVC	17 (73.91)	56 (30.9)	**<0.001**
Permanent CVC	0 (0)	2 (1.1)	1.00
Nasogastric tube	15 (65.2)	48 (26.5)	**<0.001**
Mechanical ventilation	10 (43.5)	24 (13.3)	**0.001**
**Time intervals between the SO and** **the intervention, median of days (range)**			
From SO to 1^st^ medical contact	1 (0–4)	1 (0–3.25)	0.88
From SO to intervention	11 (5–17.5)	6 (2–10)	**0.03**
From 1^st^ medical contact to admission	1 (0–6)	2 (0–10)	0.17
From 1^st^ medical contact to intervention	8 (1.5–17)	3 (1–9)	**0.04**
From admission to intervention	1 (0–7.5)	1 (0–4)	0.76
Appropriate empirical therapy (24 h), No. (%)	7 (30.4)	134(74)	**<0.001**

*IAI, intra-abdominal infection; CVC, central venous catheter; SO, symptoms onset*.

*Values in bold indicate that P-value is inferior to 0.05*.

Finally, the multivariate analysis in both the cohorts grouped together identify the patients diagnosed during the COVID-19 (*p* = 0.02), the longer period from the SO to the SI (*p* = 0.01), septic shock (*p* = 0.03), the Charlson comorbidity index (*p* = 0.001) as independent factors associated with death, and the appropriate empirical therapy during the first 24 h (*p* = 0.001) as a protective factor for death (Table 3).

**Table 3:** Multivariate analysis of the factors related to death in a combined cohort of the pre-COVID-19 pandemic cohort and the COVID-19 pandemic cohort.

**Factor**	**Death (*n* = 23)**	**Survival (*n* = 181)**	***P*-value**	**OR (CI 95%)**
Age, median (range)	72 (62–76)	63 (51–73)	0.567	1.01 (0.97–1.06)
Charlson index ≥3, No (%)	19 (82.6)	53 (29.3)	**0.001**	9.83 (4.63–17.49)
IAI patients in COVID-19 period, No (%)	16 (69.6)	80 (44.2)	**0.02**	6.23 (2.10–14.92)
Days from SO to intervention, median (range)	11 (5–17.5)	6 (2–10)	**0.01**	5.03 (2.98-8.74)
Septic shock, No (%)	7 (30.4)	14 (7.7)	**0.03**	5.69 (2.64–9.43)
Post-surgical peritonitis, No. (%)	6 (26.1)	16 (8.8)	0.705	1.32 (0.31–5.53)
Hollow viscus perforation, No. (%)	6 (26.1)	17 (9.4)	0.181	2.61 (0.39–4.47)
Appropriate empirical therapy (24 h), No. (%)	7 (30.4)	134 (74.0)	**0.001**	0.27 (0.01–0.41)

*SO, symptoms onset; CI, confidence interval; OR, odds ratio*.

*Values in bold indicate that P-value is inferior to 0.05*.

## Discussion

This study shows that the mortality of IAI by *E. coli* was higher during the COVID-19 pandemic period compared to the pre-COVID-19 pandemic period. To the best of our knowledge, this is the first study that analyzed the impact of the COVID-19 on the IAI diagnosis, treatment, and outcomes. The bivariate analysis reported that the patients with IAI during the COVID-19 pandemic had more comorbidities, required more emergency surgery, and had more severe infections. With respect to the lower percentage of the patients with bacteremia during the COVID-19 pandemic, it is due to the decrease in the blood cultures requests by the physicians and consequently less bacteremia detection in the microbiology service. According to the data, it is important to note that this study does not include bacteremia in the multivariate analysis to avoid any bias in the independent variables introduced in the analysis. Moreover, the period of time between the SO and the SI was longer for the patients with IAI during the COVID-19 pandemic period compared to the patients with IAI in the pre-COVID-19 pandemic period and it was independently associated with death.

Despite the logistical difficulties caused by the COVID-19 pandemic, no significant delay was detected between the hospital admission and the SI, a period that only includes in-hospital factors. Of note, the visit to the emergency department and the use of the operating room decreased in 2020, the COVID-19 pandemic period, in our hospital by 25 and 22%, respectively. In turn, There was an increase in the time between the SO and the first medical contact, a period that includes the prehospital and in-hospital factors and between the first medical contact and the hospital admission. Probably, these issues are owing to the time spent by the patients to go to the hospital or the primary care attention due to the necessary protection measures applied in Spain during the COVID-19 pandemic. In this study, the possible explanations for these results could be a combination of the avoidance of medical care due to the social distancing and the fear of severe acute respiratory syndrome coronavirus 2 (SARS-CoV-2) hospital transmission and to the use of the telephone consultation with the physician, a process in which the physicians had no prior experience or training.

This issue seems to be suggested in our study, in which the patients with IAI avoid going to the hospitals or they go later as reflected by the high number of patients with more severe infections. In Italy, a similar observation was reported in which the urgent emergency interventions were dropped and were associated with a more severe presentation due to a diagnostic delay (13).

In addition, this study also found that the patients during the COVID-19 pandemic had more severe infections with higher rates of septic shock and sepsis. This could be explained by the fact that during the COVID-19 pandemic, the patients were admitted to the hospital in an advanced stage of the disease. The delay in the time interval between the first medical contact and the SI could be also another factor, which could imply an increase of the complications before the abdominal surgery, which can further lead to hemodynamic instability (e.g., perforations, complicated abscesses).

In this study, it is important to highlight the worrying increase in the mortality of patients with IAI during the COVID-19 pandemic. Recent epidemiological data indicate a significant increase in mortality during this period that cannot be fully explained by the patients with the COVID-19 alone (14). Lack or delay in access to the specific antimicrobial treatment also would imply an increase in the severity of infections. Thus, it may be useful that recommendation statements devote attention to the management of the patients with IAI and the other severe infections during the COVID-19 health crisis.

This study also has several limitations. The first is the small sample size used and the patients are recruited from a unicenter cohort. A large sample and multicenter cohorts would be welcomed to confirm our results. Moreover, this study has only been able to analyze the available data in the pre-COVID-19 pandemic period and during the COVID-19 pandemic period. However, this study provides some advantages. We have compared the demographic, clinical, and microbiological variables of the patients with IAI in the two periods differing only in the presence or absence of the COVID-19 pandemic situation and also identified the impact of the days before the diagnosis and surgical interventions and their effects on the death between the pre-COVID-19 pandemic period and the COVID-19 pandemic period.

## Conclusion

This observational study showed the impact of the COVID-19 pandemic on the clinical outcome and death due to IAI with an extension of time between the SO and the SI.

## Data Availability Statement

The original contributions presented in the study are included in the article/supplementary material, further inquiries can be directed to the corresponding author/s.

## Ethics Statement

The studies involving human participants were reviewed and approved by the Ethics Committee of the Virgen Macarena and Virgen del Rocío University Hospitals (approval no. 0023-N-16 and 0088-N-20). Written informed consent was signed by all patients before inclusion in the cohorts. The patients/participants provided their written informed consent to participate in this study.

## Author Contributions

LG-B, ÁR-V, and RÁ-M conducted the study, collected data, and analyzed the study. LG-B and YS conceived and designed the study. YS wrote the manuscript. RÁ-M, RJ-R, JL-J, and JP revised and approved the final version of the manuscript. All authors contributed to the article and approved the submitted version.

## Funding

This study has been funded by the Instituto de Salud Carlos III through the grant CP15/00132 and the project PI19/01009 (co-funded by the European Regional Development Fund/European Social Fund; A way to make Europe/Investing in your future) and by the National Plan R + D + I 2013–2016 and the Instituto de Salud Carlos III, the Subdirección General de Redes y Centros de Investigación Cooperativa, Ministry of Economy, Industry, and Competitiveness, the Spanish Network for Research in Infectious Diseases (REIPI RD16/0016/0009), co-financed by European Development Regional Fund A way to achieve Europe, Operative program Intelligent Growth 2014–2020. YS was supported by the Subprograma Miguel Servet Tipo I from the Ministerio de Economía y Competitividad of Spain (CP15/00132). ÁR-V was supported by the Subprograma Rio Hortega from the Ministerio de Economía y Competitividad of Spain (CM18/00122).

## Conflict of Interest

The authors declare that the research was conducted in the absence of any commercial or financial relationships that could be construed as a potential conflict of interest.

## Publisher's Note

All claims expressed in this article are solely those of the authors and do not necessarily represent those of their affiliated organizations, or those of the publisher, the editors and the reviewers. Any product that may be evaluated in this article, or claim that may be made by its manufacturer, is not guaranteed or endorsed by the publisher.
